# Idiopathic Hypertrophic Pyloric Stenosis in an Adult, a Potential Mimic of Gastric Carcinoma

**DOI:** 10.4061/2010/614280

**Published:** 2009-09-01

**Authors:** Alireza Zarineh, Marino E. Leon, Reda S. Saad, Jan F. Silverman

**Affiliations:** ^1^Department of Pathology and Laboratory Medicine, Allegheny General Hospital/Drexel University College of Medicine, 320 East North Avenue, Pittsburgh, PA 15212, USA; ^2^Department of Pathology and Laboratory Medicine, Ohio State University Medical Center, S-305 Rhodes Hall 450 W. 10th Avenue, Columbus, OH 43310-1228, USA; ^3^Department of Pathology and Laboratory Medicine, Sunnybrook Health Sciences Centre, University of Toronto, 2075 Bayview Ave, Toronto, ON, Canada M5N 4M3

## Abstract

Primary or idiopathic hypertrophy of the pyloric muscle (IHPM) is a rare entity with uncertain pathogenesis which both clinically and pathologically mimics gastric cancer. We present a rare late-occurring case of IHPM in a 71-year-old Caucasian man with no apparent predisposing factor. Imaging studies demonstrated gastric distension with air fluid levels and no evidence of extrinsic compression. At upper endoscopy, massive gastric distension and no evidence of any ulcer or other mucosal defects were observed. Microscopically, marked hypertrophy of muscularis mucosa with smooth muscle cells arranged in whorls and fascicles was present which gradually transitioned to normal areas. The muscle fibers stained with smooth muscle actin and trichrome stain highlighted fibrosis between the muscle fibers. Although uncommon, IHPM can clinically and histologically mimic other proliferations in the gastric wall, such as gastrointestinal stromal tumor or a spindle cell neoplasm. The recent advances in understanding the pathogenesis of IHPM are discussed.

## 1. Introduction

Primary or idiopathic hypertrophy of the pyloric muscle (IHPM) in adults is a relatively rare, yet well-established entity [1, 2]. The incidence of congenital hypertrophic pyloric stenosis is reported between 0.25% and 0.5% of all live births in literature [3, 4]. The adult variant, however, is even more uncommon with less than 200 cases reported in the English literature [5]. Although, it is unclear what causes this condition, theories have been proposed such as the persistence of a mild manifestation of a juvenile form into adulthood [2, 6]. IHPM appears to be far more common in middle aged males [7, 8].

We report a case in an older male with no prior history of gastrointestinal symptoms and no apparent precipitating factor.

## 2. Report of a Case

A 71-year-old Caucasian man had been experiencing abdominal distension, nausea, and vomiting for three months. His symptoms worsened progressively over the previous two weeks when he was referred to our institution for further evaluation. The abdominal distention and vomiting appeared to be mostly postprandial, and the vomitus consisted of mainly undigested food and no bile. His symptoms were independent of the type and consistency of the type of the food ingested. His past medical history was significant for cardiovascular diseases, end stage renal disease, and diabetes mellitus with no history of gastrointestinal problems.

Abdominal films obtained before admission showed massive gastric distension. Physical exam revealed a soft, mildly distended abdomen. No tenderness, mass, and/or hernia were discovered. The remainder of the examination was unremarkable except for his dialysis fistula in right upper extremity. He was admitted with diagnosis of gastric outlet obstruction. Abdominal films following admission again demonstrated gastric distension with air fluid levels. A CT scan did not show any evidence of extrinsic compression.

**Figure 1:**
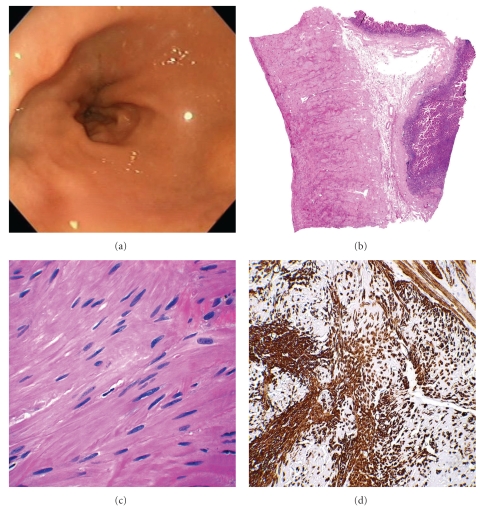
(a) Esophagogastroscopy (EGD) of the pyloric canal showing marked narrowing and failure to relax after dilatation. (b) Full mount of the pyloric region cross section demonstrating marked thickening of the muscularis propria layer (scanning magnification). (c) High magnification showing fascicles of smooth muscles with a disorderly stratification (high power). (d) Immunhistochemical stain for smooth muscle actin antibody confirms the presence of layers of smooth muscles (high magnification).

An upper endoscopy (EGD) demonstrated massive gastric distension with a large collection of partly digested food. There was no evidence of an ulcer or other mucosal defect in the pyloric channel, but the channel did not appear to relax and dilate (Figure 1(a)). The endoscope passed the channel with slight resistance, which was interpreted as pyloric stenosis. The duodenum was unremarkable.

The patient did not improve, and surgical exploration was recommended. At exploratory laparotomy, a massively dilated stomach was encountered. During this procedure, a minigastrotomy was made on the greater curvature of the stomach, and greater than 3 liters of foul-smelling particulate matter gastric contents were removed, consistent with longstanding food retention. The origin of this retention appeared to be due to pyloric stenosis with an associated thickened gastric wall. An antrectomy with Roux-en-Y gastrojejunostomy was performed, and a Witzel feeding jejunostomy tube was placed.

## 3. Pathologic Features

The specimen consisted of a segment of stomach, antrum and pylorus measuring 9 × 6 × 4 cm. The wall of the stomach was uniformly thickened over the proximal portion of the specimen involving the entire circumference of the gastric wall. A very prominent gastric fold measuring 1.5 cm in thickness and located at 1 cm from the distal margin was observed at the pylorus. There were focal areas of congestion in the mucosa, but no masses or ulcerations were seen. 

Microscopically, marked muscularis propria hypertrophy with smooth muscle cells arranged in whorls and fascicles were seen in the pylorus, while the remaining stomach also showed a thickened muscularis propria (Figures 1(b) and 1(c)). Transition between thickened and normal areas was gradual. The maximum pylorus muscle thickness measured at 1.3 cm. There were also focal mucosal changes consistent with reactive (chemical) gastropathy and a single hyperplastic polyp was present. Lymphoid aggregates consistent with mild chronic gastritis were present in the gastric mucosa. No evidence of diabetic gastropathy including hydropic neural degeneration, vasculopathy, or smooth muscle degeneration was present.

Immunohistochemistry staining for smooth muscle actin (clone 1A4, Dako Cytomation, Carpenteria, CA; 1 : 150) confirmed the thick layer of smooth muscle (Figure 1(d)). Trichrome stain showed the presence of fibrosis between the muscle fibers.

## 4. Discussion

Adult IHPM has been previously described in literature [1–15]. Patients with adult hypertrophic pyloric stenosis often have history of epigastric pain or vomiting with occasional relief after vomiting [1]. 

Radiology examination can be normal in many cases [9, 15], and endoscopy is often needed to make the diagnosis of IHPS and exclude other causes. Schuster defined a unique endoscopic sign called the “cervix sign” to describe the narrowing of the pylorus [10]. This sign is consistent and persists after anticholinergic therapy, and pressure by the endoscope differentiates it from the far more common pylorospasm [11].

Microscopic examination demonstrates marked hypertrophy of pylorus muscles which may be associated with reactive mucosal gastropathy. However, no other significant pathologic process such as inflammation or neoplasm should be seen in the muscularis propria. The transition of the hypertrophied section is usually gradual from the normal areas [9, 12]. The presence of fibrosis in addition to smooth muscle hypertrophy can often be present, as was seen in our case.

The exact etiology of adult IHPM is unclear. The most widely accepted etiologic classification includes a primary type with no apparent underlying disease and a secondary type. The secondary type can have many causes including exuberant healing of a previous gastric or duodenal ulcer, carcinoma, gastrointestinal stromal tumor, extrinsic postoperative adhesions, bezoars, and vagal hyperactivity leading to muscular hypertrophy [4, 13, 14, 16]. The secondary type usually demonstrates a predominantly localized replacement by fibrous tissue, with little or no smooth muscle hypertrophy. Other rare reported causes are postinflammatory complication in Crohn's disease [17] and mucosal diaphragm [18]. Stress ulceration at the pylorus has been proposed as the cause of infantile hypertrophic pyloric stenosis [19], although it does not seem to have a role in the adult form. The only explanation proposed for the primary type is the persistence of the juvenile form that later presents in adult life [2, 6, 20]. However, considering the age of onset of 30–60 years [7], it is unclear why the majority of patients remain asymptomatic until middle age, although it is speculated that it may be that the condition is aggravated by other gastric pathology [4]. Finally, as with infantile form, a familial tendency of occurrence has been documented for adults with hypertrophic pyloric stenosis [21], and both forms can occur in the same family. However, some cases appear to arise de novo at a more advanced age [2].

Differential diagnosis includes the more common neoplastic processes and diabetic gastropathy both can have the similar clinical presentation. Though recognizing carcinoma is often straightforward, the spindle cell neoplasms such as gastrointestinal stromal tumor might be more difficult to differentiate from IHPM. Diabetic gastropathy shows the characteristic hydropic neural degeneration with severe reduction in the density of unmyelinated axons, vasculopathy with thickening of the vessel walls, and smooth muscle degeneration and fibrosis, with eosinophilic inclusion bodies (M-bodies) which appear to be unique to this condition. 

The preferred treatment is surgery with gastric resection and Billroth I anastomosis [7], as was performed in our patient. Although pyloroplasty and vagotomy have also been performed with successful results [16], recurrence has been reported with this approach [22].

We present this relative unusual presentation of gastric outlet obstruction to raise the awareness of the existence of this lesion and not confuse the histologic findings with other proliferations in the gastric wall such as a gastrointestinal stromal tumor or other spindle cell neoplasms. Attention to the clinical and histologic features should allow a correct interpretation and prevent misinterpretation of a neoplasm, especially at the time of frozen section.
